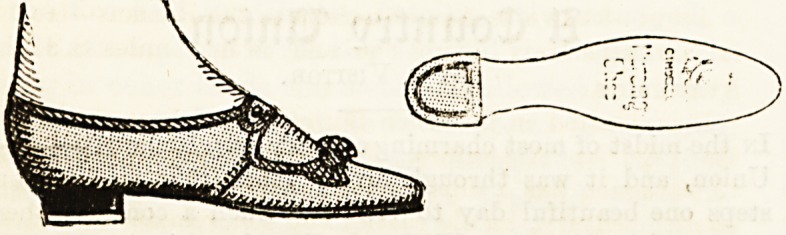# The Hospital Nursing Supplement

**Published:** 1894-05-19

**Authors:** 


### "The Hospital" Nursing Mirror.

Being the Extra Nursing Supplement of "The Hospital" Newspaper.

[Contributions for this Supplement should be addressed to the Editor, The Hospital, 428, Strand, London, W.C., and should have the word "Nursing" plainly written in left-hand top corner of the envelope.]

### News from the Nursing World.

### NURSES AND VACCINATION.

Some time ago we commented on a report of the Metropolitan Asylums Board relating to the small mortality amongst those in charge of the sufferers from small-pox in the institutions of the Board. Experience went to show that nurses who had been re-vaccinated within reasonable periods had hardly anything to fear in nursing the disease. The results of re-vaccination and the contrary are being discussed much at present in the papers. The figures are very striking, and should make all nurses who have not been re-vaccinated hasten to be so. Whilst the statistics of the Metropolitan Asylums Board showing that 655 re-vaccinated attendants escaped infection, 10, who were only vaccinated once, were all attacked by the disease. Facts from Leicester, Sheffield, and other places are no less striking. The disagreeables of vaccination are short-lived, and in face of convincing facts, even if individuals do not entirely believe in its efficacy, little harm can be done in bowing to a general and growing belief.

### GUARDIANS AND NURSES.

An amusing discussion arose out of an attempt to prevent the Kidderminster Guardians from employing a district nurse and contributing to nursing institutions as well, by one of their number. The action of the dissentient member of the Board was compared at the meeting to that of a member of the House of Commons appealing to the House of Lords against a Bill that had been passed. The dissatisfied guardian had written an appeal to the Local Government Board against the decision of the Board. The district is large, and to us the Board appear wise and public-spirited in providing that the poor should be able to obtain proper nursing. They are to pay their nurse £70 per annum, and not require her to do the whole work of the district, as the dissatisfied gentleman seemed to think that so costly an official ought to do. We hope the Local Government will not impede the excellent intentions of the Kidderminster Board to undertake a work of such undoubted benefit to the whole neighbourhood.

### DUTY FIRST.

Whatever faults have been put down to the younger generation of nurses, that of leaving the post of duty has not yet been ascribed to them. Quite recently a doctor placed a patient in the hands of "a nice old-fashioned nurse, not one of those stuck-up hospital nurses, you know." The lady was seriously ill, and being comparatively friendless, was left entirely in the nurse's hands at night. The doctor having arranged his holiday, left another medical man in charge. On the evening of the doctor's departure the nurse only made her appearance to say she wouldn't be able to come that night, as someone else wanted her. The patient, left in charge of kind but inexperienced servants, was naturally nervous and indignant, and a bad night and retarded recovery resulted in consequence. In the earlier days of her office she kept her patient amused all night, as she was wakeful, with the most interesting nursing experiences. Training may not always bring perfection, but we are inclined to think ourselves that it gives the patient a better chance of recovery.

### ASYLUM PATIENTS.

The days when cruelty to patients in lunatic asylums could pass undetected and un-redressed has long past. The public can feel increasing confidence in delivering their afflicted relatives to the care of the public institutions. At an Irish asylum rough treatment was exercised by two nurses on a refractory patient; an investigation ending in the dismissal not only of both nurses, but in a request to the nurse in charge that she would send in her resignation and apply for her pension. Patients have little to fear where discipline of the staff is so well enforced.

### A SMALL RETURN.

We learn that a District Nurses' Association has been started at Hucknall Torkard—a mining district. The idea, no doubt, is admirable of connecting the mining population with the association, but we think our contemporary must have been mistaken in stating that on payment of sixpence per annum miners could have the services of a nurse free. We imagine the sum should have been at least per month, and even then, regarded as a payment, the contribution would be small enough.

### A NEW NURSES' HOME.

A new Home for Nurses is to be built in connexion with the Infirmary at Paisley, through the generosity of Mr. Peter Coats, of the firm of J. and P. Coats, thread manufacturers, Paisley. The home is to be detached from the infirmary buildings proper, and a separate room is to be provided for each nurse. The cost of the new building is estimated at £10,000. The directors of the Infirmary are to be congratulated upon so munificent a gift.

### DISTRICT NURSES AT STRATFORD.

St. Mary's Maternity Charity and District Nurses' Home at Stratford has had special efforts made on its behalf to meet the demand upon its funds. A sale of work was held in the Town Hall, and a collection has raised over £250. The nurses become more and more in demand. The staff numbered 30 last year. It has now been necessary to increase it to 35. We are told that as many as 150 visits have been paid by the nurses in one day, and that the number of cases attended in nine months amounted to 1,435. Connected with the institution is a much appreciated diet kitchen, from which 1,000 meals were sent out during the same period. Several branch kitchens are now at work, and are presided over by the nurses, who therefore have a varied and interesting if arduous experience at Stratford. In so poor and populous a neighbourhood the nurses are obliged to supply much that rich neighbours can give to poor ones elsewhere. Dwellers in localities where few poor are to be found, might give of their abundance to the Stratford Association, knowing that the money would be well and usefully distributed where it is so much needed.

### DISTRICT NURSING AT LEWES.

At a public meeting in connection with the Lewes District Nurse Fund, recently held, a most satisfactory account was given of the work done during the past year, which is especially encouraging as it is a first experimental attempt to provide skilled nursing for the poor in Lewes. Nurse Brooker's care of the patients under her charge has met with much appreciation, and we cordially hope the Provisional Committee, who have so successfully inaugurated the Fund, will find their appeal for continued help warmly responded to. The committee wisely limit the nurses working day to eight hours, as far as possible. Much credit is due to Miss Laurence, the Matron of the Victoria Hospital and Lewes Dispensary, for her early efforts to secure for Lewes a system of trained district nursing.

### ENTHUSIASM AT KETTERING.

The interest taken by the good people of Kettering in their District Nursing Association is most refreshing. Quite lately a Church parade on behalf of the association was held. Hundreds of the inhabitants of the town bestirred themselves to take part in the proceedings, and the weather being fine, the scene was animated and enthusiasm great. Every available seat was occupied in the church, and an eloquent sermon was preached by the rector.

### UNIQUE METHODS.

To keep up an even temperature is of course the ambition of every one engaged in nursing an acute medical case. If the house be reasonably well built, and the fire and ventilation intelligently managed, a suitable atmosphere is readily secured in England. In nursing in other lands the methods followed must vary with the climate. Portugal, however, seems to be noted, according to travellers, for an altogether original custom. A patient suffering with typhoid fever, or pneumonia, lies in a close, yet chilly room, for windows are not made to open in Portugal, and neither sunshine nor fresh air gains admittance to the vault-like sleeping chambers. Therefore, the relatives of the sick, as a matter of course, invite their friends to come at once and help to warm the room! A small crowd soon collects; the door is kept closely shut, and ere long the temperature is indeed raised. The sick person no longer complains of feeling chilly, and his friends depart contented at having helped to warm his apartment in this neighbourly fashion. What wonder that disease is endemic in picturesque Portugal.

### NURSING IN GLASGOW.

The twentieth report of the Glasgow Society for Providing Nurses for the Sick Poor shows a good record of work in the past year. The society has lost a valued friend in the death of Miss Macpherson, the late superintendent and founder and organiser of the society. On Miss Macpherson's retirement last autumn, as a commemoration of her services, one of the society's vice-presidents gave £500 towards a fund to be called after her, other friends raised the sum to £1,282, and with the interest of this money new premises were secured, and a good committee-room and offices provided, where the work is now carried on with much greater advantage. Nine nurses are attached to the society, by whom 620 cases have been visited during the year. A very excellent scheme, called by its originator, Mrs. Nicholas Grimshaw, the "Sunshine Society," provides weekly half-holidays in the country for the convalescent district patients. A luncheon basket for two, and two shillings for railway fare, is given every Saturday through the summer by Mrs. Grimshaw. Lady Londonderry, the Countess of Shaftesbury, and other ladies also undertake baskets during some part of the summer months, and we hope the committee's appeal for additional help in this endeavour to "add to the health and pleasure of the poor during the coming summer" will be widely met.

### LECTURES IN MELBOURNE.

An excellent lecture on "How to Prevent Illness" has been given at Melbourne by Dr. W. Atkinson Wood. It appears to have been known beforehand that the subject of the "Rearing of Children" would receive special attention, and the large audience included a vast number of mothers with babies. Such eagerness for instruction augurs well for the practical application of the teacher's remarks, and the District Nursing Society, under whose auspices the lecture was given, are to be congratulated on it and on the other valuable work which it accomplishes so steadily.

### SAN FRANCISCO NURSES.

The nurses of the training school in connection with the Children's Hospital, San Francisco, held festival on April 2nd, when diplomas and badges were presented to the various graduates. The lecture-hall was prettily decorated, and many guests were present. Suggestive addresses to the nurses were given by the Rev. Floyd Mynard and Dr. W. E. Hopkins. The former spoke of the two years' strenuous training, of hard work and self-sacrifice, which should bear ripe fruit in future years, as a bright spot in after-memory. A very pleasant entertainment, with music and dancing, followed the presentation of certificates, and brought a happy evening to a successful close.

### SHORT ITEMS.

Miss Willoby, the late treasurer of the Berwick Ladies' District Nursing Institution, has resigned her post owing to friction caused by certain confusions between the respective duties of the secretary and treasurer.—The Kendal Home Nursing Association recently held its first annual meeting, when an extremely satisfactory report of work done in 1893-94 was given. There appears to be much need for an increased nursing staff.—The Horwich District Nurses' Association has received £27, the proceeds of a football match between the District Police and the Tradesmen.—The Wexford County Infirmary has lost the services of Nurse Nolan, who has been a faithful and competent nurse at the institution for twenty-eight years. Nurse Nolan retires with a pension.—A District Nurse is to be provided for Redruth.

### On General Nursing.

By Rowland Humphreys, M.R.C.S., L.R.C.P.Lond.

XII.—PNEUMONIA (*continued*).

The best form of reducing the fever seems to be the use of baths, tepid at first, gradually cooled to 60 deg. Fahr.; these are given for about twenty minutes at a time, watching the pulse and temperature, and bearing in mind that the temperature continues falling for half an hour after the bath is discontinued. The wet sheet does not appear to have sufficient effect to be of much use to adults, but it is very useful in the case of children, or where, as in very stout persons, the difficulty of moving them is considerable. Sponging is probably the next best refrigerant to bathing, and may be employed, like the bath, as often as the temperature rises over 103 Fahr. For this purpose the patient is stripped and wrapped in a blanket, avoiding unnecessary exposure, and is sponged in "quarters," the sponge being carried downwards in the upper halves of the body, upwards in the lower halves. The front is done before the back, and the extremities done last. It has to be borne in mind that the object of the bath is to reduce the fever in order that its effect on the heart may be lessened. Fevers cause fatty degeneration of the muscle of the heart. "The muscular tissue is flabby and friable, so that the thumb and fingers are easily pushed through the muscular wall" on post-mortem examination. Under the microscope the muscular fibres are seen to be made up of little beads of fat from end to end, the muscular substance having disappeared. The danger of this condition lies in this, that if far advanced, if much of the heart's muscle is affected, the organ may undergo acute dilatation on a very slight bodily exertion, such as by sitting up, or by even a less exertion than this; becoming paralysed sudden death occurs. Dilatation of the heart from this cause was frequently observed during the recent epidemics of influenza.

The refrigerating process is left off when the temperature has fallen to 101 deg. Fahr., or if the patient complains of feeling cold. It is discontinued altogether if much albumen appears in the urine. The sponging is best done with water at about 116 deg. Fahr., and should be employed in cases where the heart is, or has become very weak.

Ice-bags are also used to reduce temperature, and may be applied either to the chest over the affected part of the lung or in the form of an irrigator. In either form some thin material has to be placed between the skin and the ice-bag, or coil.

Sometimes an ice-cradle is used. "It consists essentially of an iron surgical cradle, from the central bar of which are suspended several small zinc pails half filled with ice. The patient lies undressed, upon the bed, and the cradle covered by a light counterpane, is placed over him." A hot-water bottle may be applied to the feet if desired.

Another way of applying ice is to lay a piece of lint or muslin on the chest and on it place fragments of ice, the water from them running into a trough formed by a mackintosh so that the bed does not get wet.

In bad cases near death the bath has little power of reducing the fever, or, at least, it is reduced with great difficulty, and rises very shortly after the bath is left off to the same degree as before. In very weak conditions, where movement is impossible, and no ice-bag or coil handy, the patient may be exposed to a current of air, the sole objection to this method lying in its slowness.

To assist the circulation when bad the administration of oxygen is nowadays frequently used; and strychnia is often administered with it. It is given from an indiarubber bag which has been previously filled with the gas from an iron or steel cylinder in which it has been stored under great pressure. The tube from the bag is brought close to the nostrils and some of the gas allowed to escape each time the patient takes an inspiration; the mouthpiece supplied with the apparatus may or may not be used. In desperate cases the gas may be given so that the patient receives it almost pure. This may be accomplished by the use of a suitable mouthpiece, or by placing the tube in one nostril and closing the other. Under its influence the pulse gets slower and stronger, and the blueness of the face goes, and the patient becomes more conscious and is better able to take food. It sometimes sets up spasm, and requires to be mixed with air in the bag. Sleeplessness may be treated either by sulphonal or other suitable sedative.

*Catarrhal Pneumonia.*—This complaint, also known as broncho-pneumonia, is almost confined to children under the age of five years. It is invariably preceded by bronchial catarrh, which first of all blocks the smaller tubes leading to the air-cells, and then these become in turn emptied of their air, either by expiratory efforts, or by absorption, and then filled with the products of the cararrh of the tubes, being followed later on by infiltration of the tissues of the lungs by inflammatory products derived from its blood vessels. It appears likely, too, that the alveolar walls, the sides of the air cells, become at first glued together by the thick catarrhal discharge, later on, in many cases, becoming united by inflammatory material.

The symptoms in these cases depend on whether the attack comes on very acutely or whether it comes on more gradually. In the former case it bears a very great resemblance to acute croupous pneumonia. It differs from it in that sooner or later several patches of inflammation are found in the same lung, and that the inflammation spreads by the increase in the number rather than in the size of these patches. There is also always a previous history of a cold "on the chest" and the age of the patient, either very old or very young, is against acute pneumonia, though there are exceptions in both cases.

(*To be continued.*)

### A Country Union.

By a Visitor.

In the midst of most charming country stands the H——— Union, and it was through lovely lanes that we bent our steps one beautiful day to visit it. Such a contrast there presented itself to us. Without, all spoke of joyous life, the trees bursting into bud, the birds singing hymns of praise, flowers springing into bud, and over all God's glorious sunshine—a day to make one glad to be alive and to look forward with hope to the coming years; within those walls, only a weary waiting for death.

In the first room we entered sat six old women doing nothing. The room was clean and bare, and a few primroses in a cracked cup gave a faint suggestion of a world outside. Our entrance was greeted with a show of interest, and by feeble curtseys and toothless smiles. My guide, Mrs. C——, is regarded as their good angel, for she spares time from her own busy, happy life to try and gladden these dreary ones. Each old woman was presented with a new-laid egg. One old woman, more bent and feeble than the rest, brought to our notice a pot of hyacinths which she was guarding with loving care. Her next neighbour remarked, "Ah! we used to 'ave flowers giv' us for the table, but we ain't had any this many a day; that one (pointing to the hyacinth) belongs to Mrs. Sands, and we don't get much good of it, for she keeps it in the yard all day, and under her bed at night." Once, no doubt, these old women were happy girls playing in the sunny lanes, then wives and mothers; now in loveless loneliness they sit waiting for death. No prattling grandchildren round their chairs, no cozy corner; only bare walls and hard seats.

Upstairs, more old women, too weakly to come down. One quite blind lay racked with rheumatism, in pain and darkness for life, and yet she could smile as she spoke of other days.

In the midst of the old women was a cradle containing a bonny baby boy, and he smiled and crowed, unconscious of the hard times in store; the women nursed him with loving care—perhaps he reminded them of their own little ones in the bygone days.

In another small room sat a younger woman minding three or four babies, the mothers being at work in the laundry. Mothers, alas, with all the cares and pains of motherhood, but without the dignity and joy of wives.

Many a girl only realises her position when she awakens to the fact that with} an infant in her arms it is impossible to find work, and henceforth for many a weary year the four walls of the union must represent her only home. Many of these girls are very young and very ignorant, but can no helping hand be held out to help them rise to better things? If "Men may climb on stepping stones of their dead selves to higher things," must girls of seventeen or eighteen be condemned to the hopelessness of life in a workhouse?

Here and there good women like Mrs C—— are doing what they can, but there is a large work still neglected. Are there no hearts and hands willing to undertakeit? Could not some scheme be set on foot to meet the needs of country unions in this special way, saving young girls from going from bad to worse in sheer despair?

### Novelties for Nurses.

### A NEAT AND DURABLE WARD SHOE.

(Messrs. D. Nicholson and Co., St. Paul's Churchyard.)

Nurses requirements are occupying more and more attention, and now the difficulty is not where to go to obtain the most ordinary needs of uniform, but what to choose amongst a constantly improving selection. Nurses have very varied tastes, though they are much restricted by their calling from exercising them. Manufacturers are determined they shall feel the restriction as little as possible. If one firm brings out a broad and plainly-made shoe another provides one a little more dainty and pretty. Messrs. Nicholson have certainly succeeded in offering nurses a shoe which is really neat, becoming, and durable. The shoes have, of course, the necessary indiarubber heels, and are quite suited to hard wear. They are of very moderate price—5s. 11d. the pair—which wearers will admit is a small sum to give for shoes that will last. The strap over the instep admitting of adjustment is an extra advantage for nurses.

### Ladies in Australia.

### FEMALE FACTORY INSPECTORS.

Miss Margaret Gardiner Cuthbertson has been selected from about one hundred applicants by the Public Service Board of Victoria to fill the newly created position of female inspector of factories. Victoria leads the way in this matter, none of the other Australasian colonies having yet made this step forward. Miss Cuthbertson has been nearly six years in the Civil Service, in the Telephone Department, which ranks as non clerical; before that she had been for about five years in the employment of Messrs. Sands and McDougall,\wholesale stationers, and had been charged with the duty of drawing up the annual report required by Government of the female divisions of their workshops.

### Women's Work.

### CHOICE OF A PROFESSION.

Choosing a profession is a far more serious and difficult matter than appears at first sight. It does not do to pick up first one thing and then another. As a general rule each must abide by her primary choice, for the knowledge of having mistaken our vocation comes mostly too late to be remedied. If a girl is able, or thinks she is able, to throw aside her occupation at will, she seldon gets on in life. For many girls, those who must work, it is most difficult to decide what line to take up, because they have seldom the means to follow their own inclinations for painting, sculpture, music, and the like. What is left for them, then? There are girls who have just left school—laughing, bright, giddy, and thoughtless; and they are told they must do something for themselves soon. Perhaps they laugh about it, or, trying to be serious a moment, think what they really should like to take up. Finally they discover that they have not two ideas on the subject. Some become nurses in the end, and may prove that they are the right girls in the right place. But, alas! not always is this so. However, women are now permitted to turn if they like to almost any profession nowadays, which speaks volumes for their patience and perseverance; they have had hard battles enough to fight against pride and prejudice. There are portions of a woman's brain which are not developed because never brought into active play. Nothing can grow that is not exercised or made use of, and it stands to reason that we gradually lose the use of any portion of the body which we allow to remain dormant or inactive long enough.

Some time ago a lady suffering from hysteria was unable, as she believed, to move either her right arm or leg. When seen a few months later both limbs were found shrunken to one half the size of the corresponding ones, which she had constantly used. In a convict prison in Australia the convicts were all standing in rank after their mid-day meal, previous to being dismissed to their various works. The governor pointed out three who had formerly been bank managers. Thinking of the stone quarries where they were employed, a visitor inquired, "Is not this terrible work for men accustomed to every luxury?" "Yes," answered the governor. "It is dreadful for them at first! They return each night half dead to their cells, but when once their muscle begins to grow they do ten times the work of ordinary mechanics for they bring all their superior sense and will power to their aid."

And the same is true of brain as well as muscle. They both improve by being used. When a girl finds herself compelled to earn her own living there are then several practical points to be considered. She must not ask herself "Which is the easiest profession?" or the lightest—least work and most pay—but rather, "Which kind of work am I most suited for? Into which can I enter heart and soul?" Vague ideas are quite useless; they must be strictly practical. Whatever career be embarked upon will mean hard work, not play. Therein is much comfort, for regular work brings happiness if undertaken earnestly. For example, it is not an easy thing to be a nurse—far from it; it involves much hard work, patience, and self-sacrifice; therefore only those who love nursing for nursing's sake should dream of entering the profession. It is not everyone who is fitted for clerk's work; it requires a quick, clever brain; nor can any hand wield a journalist's pen, or all fingers ply a typewriter's machine. In taking up what is intended for life-work, not only "an interest" but a keen pleasure in it is needed to ensure success, whether medicine be in question, art, literature, nursing, massage, typewriting, clerking, or anything else. Therefore choose cautiously, and having chosen a profession or trade, stick to it; be content with nothing short of excellence.

### Nursing in the West Indies.

By a Traveller.

Necessity is the mother of invention, and even a temporary residence in the West Indies compels one to be to a great extent, self-reliant in the matter of nursing, or dependent upon the scanty knowledge of those who may happen to be within reach. The climate, particularly in certain districts, is very distressing, and calculated to undermine the constitution and foster disease. In the face of this fact, and one might almost be tempted to say, in defiance of it, doctors are difficult to reach—and then not always the most competent—whilst trained nurses are scarcely known. The natural outcome of this condition of affairs is that serious cases of illness are frequently placed into the hands of old native women who profess to understand the proper treatment, but whose "little knowledge" often proves itself to be a very dangerous thing. Some of them have unquestionably discovered some very remarkable remedies for complaints peculiar to the climate, but there are many others who trade upon the reputations of the few, and the credulity of the natives. As instance of this a case may be quoted of a young married woman who was suffering from weakness which the oppressive climate served to aggravate. Medical advice meant the dispatch of a messenger a distance of seven or eight miles, and the more difficult obstacle of a guinea fee. So the poor woman asked the advice of the old natives of the district. Like many better qualified practitioners, they disagreed as to the treatment required, but finally decided to combine their various opinions. Doses of pills, jalap, senna, castor oil, and other remedies were administered, and the result may well be imagined. The patient was reduced to a condition of complete prostration, and the advice of a missionary was sought. This excellent man, as became his calling, had acquired a certain knowledge of medicine, and seeing the serious condition to which the foolish treatment had brought the woman, he immediately sent off to the nearest doctor. It was unfortunately too late, and the patient, whose weakness in competent hands might well have been nursed into strength, succumbed a victim to ignorance. The missionary in question, in addition to his usual offices, which were many and various, acted for years in the capacity of medical adviser to the natives in his district, treating as many as a dozen cases of illness before breakfast. But his limited supply of drugs was soon exhausted, and it was not in his power to meet the incessant demand for them. He was not allowed, for lack of qualification, to charge a fee for treatment, by which means he might have considerably added to a small income. He was not able for lack of means to continue the system of gratuitous attendance, and was reluctantly compelled to give up the work so far as the administering of medicine was concerned.

The climate of the West Indies, with its long drought and welcome but trying spell of continuous rains, is the cause of an enormous amount of indigestion, neuralgia, and kindred ailments, particularly to those who have not become inured to it from childhood.

These complaints, trivial as they may appear to be at first thought, are at the root of a great deal of more serious and confirmed illnesses. They produce weakness and depression, and render the sufferer a ready victim to many diseases which might have been avoided had the system been in a sufficiently healthy condition to repel them. One of the most fatal of these is of course yellow fever, which particularly haunts the district of Demerara, where the want of trained nurses is most keenly felt. A case of this terrible disease recently came under the notice of the present writer, the sufferer being a young Englishman of strong constitution, but who was in a condition of complete prostration as the result of a very severe attack. The native nurses had pronounced the case entirely hopeless, and begged an English lady, who expressed her intention of endeavouring to nurse him through it, "to leave the poor fellow alone and let him die peaceably." She was determined, however, to make some endeavour to nurse him through it, and unremittingly she devoted herself to the case. She had great faith in a lotion of special preparation, and with this she daily sponged him, frequently leaving the sick room with great yellow patches of stain upon her light dress where the patient had leaned against her during the operation. The doctor seemed amazed at her perseverance, for the native value of human life is very low, and if a case is deemed hopeless it is practically abandoned. However, this patient slowly but surely mended, and eventually entirely recovered. The nurses that are wanted in the West Indies are women possessed of fearless determination to do battle with the terrible diseases which are prevalent throughout all the islands. English women are surely specially adapted for carrying on a fight against sickness, and for showing their belief that "while there is life there is hope," however discouraging the outlook.

### Acqui and its Mud Baths.

Some years ago circumstances led me to join a friend at the Mud Baths at Acqui, which have been known since the time of the Romans, although little is heard in England about them.

Baedeker in his "Northern Italy" mentions the town of Acqui as well known for its mineral waters, but says not a word of the mud and sulphur baths so efficacious for the cure of paralysis, gout, &c. Chambers' "Encyclopædia," and other authorities, just mention the place, but say little further. In Annandale's "Modern Cyclopædia," mud baths are spoken of as a kind of bath connected with some mineral springs, consisting of mud transfused with saline, or other ingredients, in which patients plunge the whole or portions of the body, and the baths of St. Amand, Barbotan, Lourdes, &c., are cited, but not Acqui. Since it is rare to find anyone who has heard of the place, I think perhaps a short account of the wonderful cures effected there may be considered interesting.

Acqui—known as Aquæ Statiella to the Romans—is a town in Northern Italy about one and twenty miles S.S.W. of Alessandria.

It is situated on the river Bormida, and is on the north of the Ligurian Apennines. It boasts a population of over 12,000 souls, has a fine Gothic cathedral dating from the twelfth century, an old castle, and the remains of a Roman acqueduct.

It is in direct communication with Alessandria to the north and Savona to the south, and is within easy distance of Turin, Milan, Genoa, and Nice, consequently accessible from any part of Europe. It possesses special advantages as regards climate. During the summer cool breezes from the mountains moderate the temperature and render the great valley genial and bracing.

There are four separate bathing establishments, three of which are at some slight distance from the city, "beyond the Bormida," a military hospital and one for the poor, kept up by the State, and the municipal hospital, with its baths, bed-chambers, dining hall, ball and concert rooms, for people of means. The municipality have also erected another large building in the city, so that the public can now be accommodated all the year round. The summer residence used to open in May and close in September, and patients stayed from 15 to 20 days at a time. There is an old saying that to be perfectly cured you must visit Acqui three successive seasons, *i.e.*, twice a year—the first time to take the baths, the second time to be cured, and the third time out of gratitude and to complete the cure.

Close to the summer establishment are some huge deep reservoirs, some quite full of the black, bubbling mud, others half-full, and some almost empty. There are hot springs beneath, which give 98 gallons a minute at a temperature of 115° to 140° Fahr. They carry in solution a considerable quantity of vegeto-mineral matter which, when precipitated, forms the mud. Near to these mnd pits are some equally large sulphur reservoirs. A narrow pathway raised some short distance above the level of the mud enables you to walk round these, but if persuaded to do so, you hurry along with a shudder, for a slight slip might land you into the horrible looking stuff beneath, and out of that you would not be taken alive.

The mud is taken out by means of long-handled wooden scoops, placed in wooden pails, and carried quickly into the different bath-rooms, which are all on the ground floor, and close to the reservoirs.

The patients come down, or are carried from their bedrooms to the bath-rooms, where they are placed of reclining wooden boards, and the whole or part of the body is quickly covered over with the hot, thick mud. I saw some curious looking wooden receptacles in the smaller bath-rooms, which are used when only one limb requires treatment.

After this hot application has been on for some time it is taken carefully off, and the invalid is usually given a warm sulphur bath, and finally a bath of clean water. If suffering from total paralysis he would retire to his bed or couch and rest—sleep if possible—until it was time to go downstairs again and have the same process renewed. Experience seems to prove that these baths are an almost infallible remedy for paralysis, neuralgia, chronic gout, rheumatic arthritis, scrofula, chronic diseases of the skin, wounds, ulcers, and sprains.

There are 100 different bath-rooms—forty for water and mud baths, the same number for ordinary sulphur baths, "Boudoirs de Luxe" for water and mud baths, and small rooms for partial application of the mud. Then there are other rooms for vapour, electric, shower and needle baths, &c. The number of baths to be taken during the day are at the discretion of the doctor, some patients, in extreme cases, having to undergo the process three times.

The number of patients is now increasing yearly. In 1842 the number was 439; in 1872 the total number was 1,398, thirty-five of whom were English, five from Africa, one from Brazil, the greater number naturally being from Piedmont and Lombardy. The winter establishment is in the most healthy part of the city of Acqui. It was opened ten years ago. The mud is brought every day from the reservoirs at the older residence in iron vessels. There is a boiling spring in the city, La Bollente giving 118 gallons per minute at the temperature of 165° Fahr. The vessels are placed in this water till required.

### Where to Go.

Women's Trade Union League.—A demonstration will be held on Thursday, May 24th, at the Memorial Hall, Farringdon Street, for the purpose of demanding the retention of certain clauses affecting laundries in the new Factory and Workshops Bill. The chair will be taken by Mr. John Hutton (Chairman, London County Council).

Royal Hospital for Children and Women, Waterloo Bridge Road.—A special concert on behalf of this hospital, under the patronage of H.R.H. the Princess of Wales and H.R.H. the Duchess of Albany, will be given by the Strolling Players' Amateur Orchestral Society, at Queen's Hall, Langham Place, on Wednesday, May 23rd, at half-past eight p.m. Tickets may be had of Mr. R. Garrard Kestin, Secretary, at the Hospital; or of Mr. R. Newman, at Queen's Hall.

### Thoughts on Training.

By an Old Nurse.

The noblest profession, as many think, is that of nursing, and yet how it seems to be often looked upon as a mere matter of self-aggrandisement, self-pleasing, or "a step up in the world." Does not training too often mean the extermination of courtesy, tenderness, and true sympathy? Even where these qualities remain, do not laziness, selfishness, and love of admiration grow more rankly in hospital soil? The trained sister or charge nurse oppressing her fellow-workers by virtue of her position. So few can wield power graciously, and the probationer of last year, so meek, tearful, and subservient, becomes, with a change of dress and work, a veritable tyrant. How many are content with being good nurses? The aim is frequently to secure plenty of testimonials and advancement to paying posts. Our smaller country charge posts are often taken by women who, with a feeble staff of two or three probationers, endeavour to act as grand ladies, filling their wards with plants and flowers (although there is no harm in these if the head nurse looks after them), and imitating in minor chords the notes struck in London wards where they may once have occupied subordinate positions. The true unselfish earnestness of sharing fairly the work of their wards is almost unknown. In nearly all hospitals it is the probationers who are the burden carriers, and the smaller the hospital the greater the load carried. Head nurses may be rude, ill-tempered, untidy, but woe to the probationer who shows off similar propensities. Ladies who in their own homes would be courteous alike to equals and servants think it no harm to be rudely masterful in hospital work. How many times a day, through the head nurse's forgetfulness, does not the tired probationers uncomplainingly go to dispensary, porter's lodge, or doctors' rooms? Does the head nurse ever think of the needless labour laid upon her weary assistant? Of course there are many wise, high-minded, courteous women, ladies in the highest sense of the word, incapable of speaking to others in a tone they would not receive themselves, women who have not forgotten "He that is greatest among you let him be your servant." True "Sisters"—willing to share in all the labour, and at all times the kindest as well as the best of the nurses in the ward. The quaint old term "Sister" carries with it remembrance of the affectionate unselfishness of the elder daughter of the home. The server, not the served—but in hospitals, particularly the smaller ones, "Sister" means often a conceited, uppish head nurse, with little real personal power, knowledge of the world, or education, but yet, by virtue of her office, she oppresses her probationers, provokes her subordinates, and grieves her superiors. Nowaday one would suppose hospitals were built for nurses—not nurses for hospitals. Such comforts surround us we shall soon surrender the old ideas of devotion and self surrender. There are grand exceptions, but who will correct me when I say the majority of trained nurses are wanting in tenderness, loyalty, and courtesy? They know their business, are apt enough at going round, operations, show work, but is it not a pity this very knowledge has trained out their highest attributes? It is good to rise in one's position, but the profession of a nurse has meant in the past higher things than the making a living and a comfortable post where others do the work. It may be many years ago, but "Sister" was once the embodiment of dignity, power, and strength, stern, perhaps, unflinching in having things well done, but never mean, or small—a character to live up to in every sense of the word. We say "probationers are not what they were." Is it not because the standard set before them has been so terribly lowered?

### Our American Letter.

Last month took place the annual graduating exercises of the Mills Training School for Male Nurses in connection with the Bellevue Hospital, New York, when fifteen young men at the completion of their two years' course received diplomas. Dr. George B. Fowler, the chairman of the board of directors, before presenting the certificates, gave the candidates a useful and practical address upon their future duties as nurses in private families. The question of male nursing is being somewhat discussed just now in the pages of our American nursing magazine, *The Trained Nurse*, in the May number of which will be found letters dealing with the subject from different points of view.

Although not yet entirely finished, the new St. Mark's Hospital, New York City, has already admitted many patients to its wards, the demand for hospital accommodation being very great in the surrounding district. Applications for admission are being received all day. The internal fittings and arrangements are all that can be desired.

The thirteenth meeting of the Nurses' International Association has lately been held at the headquarters of the association, Well's Memorial Library, 987, Washington Street, Boston. Nurses are, however, so very busy just now that the attendance was a small one, so small indeed as not to form a quorum, and those present waived ceremony and took part in an informal discussion as to the advancement of the best interests of the association. A feeling is abroad that so far the meetings have not been sufficiently social in character, and a proposal has been made to admit honorary members not necessarily nurses.

The hospital of the University of Pennsylvania is badly needing financial help at the present time to enable many essential improvements to be carried out. Increased accommodation for patients is wanted, and a new laundry, while the maternity hospital is waiting completion, and the needs of the hospital in these directions has been urged upon the state legislature with the approval of the state board of charities, with the result that an Act has been passed to secure 60,000 dollars for the erection of new hospital buildings and 20,000 dollars for the new maternity block, but these sums are not to be available until such time as a further 80,000 dollars shall have been contributed from other sources. These are bad times for such an appeal, but the managers earnestly hope the friends of the hospital will come forward and make it possible for this provisional grant to be used. The authorities are particularly anxious to substitute electric lighting throughout the hospital for the gas in use at present, but the funds for so doing have not been hitherto forthcoming.

A very good and useful work is being done through the "babies' ward" of the Post Graduate Hospital, New York. The little ones are admitted regardless as to the curability of the case, with the humane desire to at any rate render their last days as comfortable as may be. The report of Miss Ella Russell, the chairwoman of the ladies' auxiliary committee, will arouse much sympathy.

The Chicago, Milwaukee, and St. Paul Railway Company have been proposing to establish hospitals for its employés at the principal towns along its lines, but the members of the American Railway Union have voted heavily against the plan.

The Children's Hospital, Ottawa, has been having a struggling year. The old building having been found both too small and inadequate in many ways, a new wing has been erected and other additions and alterations carried though. The new Lady Superintendent, Miss Alice Stone, has proved herself invaluable, and has devoted her energies and her money to the successful completion of the work. The new building is now all that can be desired, most home-like and comfortable, and the nursing staff is thoroughly efficient. Two of the nurses are experienced masseuses.

At the request of certain of the medical staff of the Children's Hospital, Boston, the Instructive District Nursing Association have arranged to retain the services of one trained nurse especially to care for crippled children in their own homes. A Boston lady has generously undertaken to defray the entire cost, and the money has been paid to the association.

The second annual meeting of the Alice Fisher Alumnæ has recently been held at the Philadelphia Hospital Training School for Nurses. Miss Smith, the president, was in the chair. A form of constitution was drawn up and adopted, the object of the association being stated to be "to advance the standing and interests of the graduates of the Philadelphia Hospital Training School; to extend aid to those in trouble and sickness; to promote social intercourse and good fellowship amongst its members; and to raise the standard of nursing to the highest plane."

On March 31st a pleasant gathering took place in the Administration Building of the New York Hospital, when the graduating class of the Training School for Nurses gave a reception. The building was formerly one of the mansion houses of the old city, and the large rooms were fairly filled by numerous guests, who witnessed the awarding of the badges and diplomas with much interest.

Death has made two gaps in the nursing world in the States since during the last month. Miss Rose E. Deysher died of pneumonia on March 25th. She graduated with high honours last year at the Medico-Chirurgical Hospital, Philadelphia, and was attending a case in the city at the time she was taken ill. Miss Ella W. Adams, a valued and loved member of the New York Training School for Nurses' Alumnæ Association, also died suddenly on April 5th.

Fifty-six nurses graduated at the Johns Hopkins Hospital, Baltimore, during the last year. One hundred and sixty-one applications for posts in the Nursing School were received, being an increase of forty-eight over the preceding year. Some important additions to the hospital are contemplated, notably the enlargement of the room in the dispensary, now in use for consultations. A children's and a lying-in ward are also needed.

Several American medical institutions have been enriched by the will of the late Rev. W. C. Moseley, of Newberryport, Mass. A legacy of 50,000 dollars is bequeathed to endow a Professorship in the Howard Medical School; the Massachusetts General Hospital receives the sum of 20,000 dollars; while the Boston Lying-in Hospital, the Perkins' Institute for the Blind, and the Anna Jacques Hospital each receive a bequest of 10,000 dollars.

### Presentations.

On Friday, May 11th, the probationers of Addenbrooke's Hospital, Cambridge, presented their Matron, Miss M. N. Cureton, with a handsome Oriental brass tea-tray and inlaid stand as a slight recognition of her unremitting care and kindness to them all, and of the unceasing interest she takes in their welfare. An architect of eminence writes to point out that the accommodation for the nurses and probationers at Addenbrooke's, and especially the latter, is to his mind "abominable." This presentation must be taken, therefore, as a very special testimony of real value to the devotion of Miss Cureton to the discharge of the important duties which devolve upon her. No doubt a new nurses' home, planned by some one who really understands how to design such a building, will be as readily provided by the committee as the new floors and drainage were provided.

### Appointments.

The Falmouth Hospital.—Miss Lena Cunningham has been appointed matron of this hospital. She was previously matron of the Cottage Hospital at Brixham, and also of that at Exmouth. She was selected for her present post out of 60 candidates.

### A Visit to Canada.

By Sister Norma.

### I.—THE PRAIRIE.

My mission to Canada was to pay a semi-professional visit to an old school friend who had gone in for matrimony and emigration about the same time as I had adopted the nursing profession. The health of both of us had suffered since then, so I gladly accepted her pressing invitation to act as nurse and companion to her for the next two years. Before her marriage she had been accustomed to the luxury of a house in Prince's Gate, and the devotion of indulgent parents; since her marriage she had lived in a log house on a large prairie ranch, two days' journey from any neighbours; in fact, a more desolate and a barer spot could not well be imagined. The maid whom she had taken out with her married a few months after arrival, and at the outset of my visit the only servant, "a Heathen Chinee," eloped one dark night with all the silver spoons.

Captain C——'s brother, an unsuccessful stockbroker, had joined him in the ranch, and they were both paying dearly for their ignorance of colonial farming. The handsome ex-Guardsman was now an anxious, hard-working ranchman, and his wife—my once fastidious friend—was wearing the remains of her four-year-old trousseau, and they both looked ten years older. They assured me that they were considered quite "dressy," as the husband still shaved every day, and she curled her hair! No wonder that they looked anxious, for after two bad seasons we were just entering on what promised to be a long drought. They had sunk twelve artesian wells but no water was forthcoming, and during that awful summer my heart ached to see them searching the unbroken blue of that prairie sky for a little cloud in token of rain, and this when their baby was only one week old. It pained me to see the wife's pretty parched face eagerly watching me economising the one cupful of water reserved for the baby's bath. Once a week a buckboard with a bullock team was sent to buy a barrel of water from a neighbouring ranch. It was such a small quantity, and we felt in honour bound to keep it for the mother and baby. Claret and beer and most other drinkables could be procured for ourselves by paying heavily for them in the nearest "city," as each small village is called in the West. The very thought of fresh running water makes one desperate when the only water one has seen for months is bought and drawn carefully from a barrel. Of course, rain was sure to come in abundance, but, perhaps, not until too late; the prairie fires had already burnt up every blade of grass. In her letters home my friend's descriptions of hardships had been intensely amusing, but the experiences themselves must have been most pathetic. Their weekly washing done by her husband and herself in tubs placed side by side on the verandah did not sound very dreadful to a hard-worked hospital nurse, but the reality, including the climate, was a cruel one. Now, they had not washed clothes for weeks!

The tremendous heat compelled us to remain in the house all day; we kept the little log hut comparatively cool by thick awnings over the verandah, but even in the evening there was no pleasure in going out; the prairie soil was dry and black, and rose up in clouds of dust around, and the atmosphere was thick like a London fog, with a plague of grasshoppers. There was not a breath of wind, and they flicked their heavy dust-laden wings in your face, and your dress was covered with them. For the first time I realised the Bible meaning of the grasshopper becoming a burden. I tried to picture this scene during heavy rains, but it seemed as though they could never come. On the dead level of the prairies, with no rising ground within reach, the horizon is so limited that it affects your imagination. We grew literally home-sick for green England. When at last Captain C—— grew alarmed for his wife's health, and told us we must go for two months to Banff, in the heart of the Rocky Mountains, where are the hot sulphur springs of Canada, we fairly cried. We had two days' journey across the prairie to the railway station and two days more on the cars before we exchanged the parched prairies for mountains, rivers, and canyons. It was late at night when we boarded the cars on the Canadian Pacific Railway, and most of the passengers were asleep in their comfortable berths, a few eager tourists had turned out on the platform to spy out the nakedness of the land. We played like children for an hour with the fresh water conduit in the ladies' lavatory. It seemed almost a sin to let water run off after merely washing a baby in it, and the ample hand basin made a capital bath for him. We were known as Nos. 31 and 32 in the sleeping cars, and as we followed the pompous nigger porter to our berths at the posterior end of the car an elderly crumpled bald head in one berth, or a fair young girl's from the next, or a sleepy sunburnt face with heavy moustaches from another would, from behind pushed backed curtains, take a peep at the fresh arrivals. They were easily satisfied, "only two tired women and a baby." On the morning of the second day I was awakened by a stream of sunlight forcing its way through the tapestry blinds of the window close to my head; it was a new dazzling sunlight full of fluctuations and shadows, not the old leaden prairie glare. I let my blind fly up with a bang, and found we were literally crawling round the side of a precipice, and higher than I could see out of my little window were snow capped mountains mixed up with the great masses of fast moving clouds; what a delight those armies of flying clouds were for the next two days after our skies of hard unbroken blue. The water in the gorges below from the glaciers overhead were turquoise blue, and roared like dull thunder, and every now and then would throw up clouds of white spray from some mighty cataract below.

### Notes and Queries.

The contents of the Editor's Letter-box have now reached such unwieldy proportions that it has become necessary to establish a hard and fast rule regarding Answers to Correspondents. In future, all questions requiring replies will continue to be answered in this column without any fee. If an answer is required by letter, a fee of half-a-crown must be enclosed with the note containing the enquiry. We are always pleased to help our numerous correspondents to the fullest extent, and we can trust them to sympathise in the overwhelming amount of writing which makes the new rules a necessity. Every communication must be accompanied by the writer's name and address, otherwise it will receive no attention.

### Queries.

(59) *Liverpool Hospitals.*—Kindly tell me which hospital is nearest to Earle Road, Liverpool.—*Armanda.*

(60) *The Annual.*—Does Mr. Henry Burdett's "Hospital Annual" give addresses of various hospitals in the United Kingdom and Colonies? She is desirous of obtaining foreign appointment. Also where the book may be procured.—*Old Subscriber.*

(61) *Asylum News.*—Can you tell me if a journal to be styled *Asylum News*, mentioned in The Hospital of January 20th, has yet appeared?

(62) *St. Keneleu's.*—Can you give me the address of Dr. G. W. Hambledon, late of 23, York Street, Portman Square?

(63) *District Nursing.*—I want to know where I can receive two or three months' good training in district nursing. I have had one year's hospital training. Or, failing that, the same time in some good general provincial hospital.—*Cricket.*

(64) *Properties of Foods.*—Will you kindly inform me if a book can be obtained containing the different properties of foods?—*H. M.*

### Answers.

(59) *Liverpool Hospitals (Armanda).*—The nearest hospital to Earle Road is Smithdown Road Workhouse Infirmary. The nearest general hospital is the Royal. You could soon get a list of them from a Liverpool directory, or even from the nearest police station.

(60) *Old Subscriber (The Annual).*—"Burdett's Annual" contains the information you require. The book may be obtained at The Scientific Press, 428, Strand, W.C., or through any bookseller.

(61) *Asylum News.*—No; it is under consideration, and notice of the final arrangements will appear in our columns in due course.

(62) *St. Keneleu's.*—Dr. Hambledon's address is given in the "London Medical Directory" for 1894 as 19, Bentinck Street, W.

(63) *District Nursing (Cricket).*—Write to the Superintendent, Metropolitan and National Nursing Association, 23, Bloomsbury-square, W.

(64) *Properties of Foods (H. M.)*—"The Art of Feeding the Invalid," price 3s. 6d., post free, from the Scientific Press, 428, Strand, may meet your requirements, or write for their catalogue of books, which contains several of the description you ask for

☞ For Everybody's Opinion, Reading to the Sick, Book World for Women and Nurses, &c., see page lxix. et seq.

### The Muses' Looking Glass.

### THE DEFENCE OF COSMETICS.

"No martyrdom, however fine, nor satire however splendidly bitter, has changed by a little tittle the known tendency of things. It is the times that can perfect us, not we the times, and so let all of us wisely acquiesce in the dance."

It is at all events a sign of the times that a mere boy like Max Beerbohm, nephew of the well-known actor, Mr. Beerbohm Tree, should attempt to preach us this insane doctrine, and that a new quarterly should publish the fantastic nonsense in its very first number. No doubt the editor knows his public and that the title of such an article is a great attraction to the female portion of it who have hitherto indulged in the use of cosmetics undefended. This article persuades them to consider themselves workers in a good cause; they are but aiding "the times to perfect us." "See," says the young enthusiast holding up his hands with horror, "we need not look far back to see women under the direct influence of nature. Our grandmothers . . . came out into the daylight once more and let the breezes blow round their faces and enter sharp and welcome into their lungs. Artifice they drove forth, and they set Martin Tupper to rule over them. A very reign of terror set in. Old ladies may still be heard to tell how, when they were girls, affectation was not; and if we verify their assertion in the light of such authorities as Dickens we find it absolutely true. Women appear to have been utterly natural in their conduct—flighty, gushing, blushing, fainting, giggling, and shaking their curls—they knew no reserve; no thought was held too trivial, no emotion too silly to express. To Nature everything was sacrificed. In those barren days what influence was exerted by women? Yet if the women of those days were of no great account they had a certain charm, and at least they had not begun to trespass upon men's ground. Far more serious was it when they became enamoured of rinking, archery, and galloping. Swiftly they have sped on from horror to horror; tennis-courts, golf-links, tricycle, and typewriter are but preliminary steps to the victorious occupation of St. Stephen's. But stay! these horrific pioneers are doomed, and though they scream victory none follow; for Artifice, that fair exile, has returned. Surely women will welcome their great protectrix; for upon her all their strength, their life almost, depends. Artifice's first axiom is repose. Bodily activity makes powder fly and enamel crack. Woman on her couch is a goddess; but so soon as ever she puts foot to the ground, lo! she is the veriest little sillypop, and quite done for. She cannot rival us in action, but she is our mistress in the things of the mind. Let her be content to remain the guide, the subtle suggestor of what *we* must do, the strategist whose soldiers we are. Artifice bids them wear a painted mask, behind it their minds can play without fear, they become important as in the days of the Roman Empire or as our own Elizabeth. Their faces will not become lined with thought; beautiful and without meaning will be their faces. Too long has the face been degraded to a mere vulgar index of character or emotion. Artifice demands long hours of homage, and when the toilet table is once more laden with the fulness of its elaboration we shall hear no more about proper occupations for women." We have summarized the lecture in the author's own words before embarking on a few words of comment of our own. Apart from his ethics, we find the author's illustrations and examples absurdly inaccurate. Firstly, he maligns our grandmothers. Read other novels besides those of Dickens. Dickens wrote of the bourgeoisie, of city merchants, small shopkeepers, and the denizens of slums. He seldom treats of the *beau monde* to whom we gather Mr. Max exclusively appeals. (For surely he does not advocate cosmetics for the cook and housemaid! At what hour will Beauty be able to sip her early chocolate or procure her bath if cook is enamelling her face; or how shall the housemaid sweep for us without putting foot to the ground?) Dickens draws his Lady Dedlock in *Bleak House* a person of very much account indeed, ruling every one around her. Were women ever better flattered than by Disraeli, who assigns the very highest importance to their power in politics, just as Anthony Trollope, another writer of those days, describes their unlimited influence on all things, clerical, political, and social. As models of repose Roman dames and Queen Elizabeth are cited. It is a great compliment to the author's female relatives that he should have penned this little skit. Had his mother and sisters been in the habit of spending hours at their face washes instead of pouring out his breakfast tea or interviewing the housekeeper, it is pretty certain that he, like other men, would cry out. To be able to stare for long hours later on in the day at their "beautiful but meaningless faces" would scarce compensate any man for inferior food. If women are to show no emotion what a holiday they will have! Think of the sympathy a man is always asking women for; think of the heroic way in which women smile at poor jokes and laugh at stale stories. They know no one else will do it, so—and it soothes the soul of a man to feel that he is appreciated by his own womenkind—they dissemble, and thus humour him into good temper. Indeed, women require no "painted mask behind which their minds can play." They do not need, like the actress cited, to conceal rouge in the right palm and powder in the left to dab the cheeks quickly when desiring to portray joy and sorrow. They can, if they choose, generally contrive to look cheerful and unconcerned though racked with pain or tortured by emotion; and surely the look of sympathy, followed by the convincing tear in the eye, is never beyond their contiving if they think the cause worth it. Master Max is obviously very young, and has quite naturally but a limited experience of women. All young men are captivated by beauty of feature and colour; and how many of them have lived to curse the fact. Is there a more dreary companion than the elderly woman who "was a beauty once." Like Kipling's "Mulvaney," who "was a corporil onst," she never lets you forget it. The unfortunate man who married her for her beauty only is made a slave to her selfish caprices, and the "little ways" which were so fascinating in the height of her charm are ludicrous in old age. When Mr. Beerbohm is a little more mature he will appreciate better the charms of a companionable rather than a merely beautiful woman. Happy he if a union of both fall in his way. At present, no doubt, he desires to do all the talking—the modern young literary person generally does. He has no other capacity, as a rule; and if he has he tries to conceal it. Manliness, as it used to be understood, is left "to a brutal soldiery" and "an inefficient navy," who have no time to waste over paradoxes while they are studying life as it is (and not as the idle would make it) in such elementary places as India, Burma, and West Africa. Nevertheless, he protests that women cannot rival him in action. To cite a contrary example, let him read of the two heroic nurses who accomplished the walk to Buluwayo, which feat had not then and has not since been accomplished by men seasoned to that fever-stricken country. Where would many gallant fellows now lie buried if Sister Eva and her companion had taken thought for their complexions or the lines that hardship would engrave upon the faces which for many a weary month after breathed hope and courage to the dying, and kept alive the ideal of true womanhood among the hundreds of rough men who crowded that pioneer city? Moreover we believe that even society dames are finding out that a far finer complexion is to be obtained by attention to health, diet, and exercise than can be produced by the most costly of cosmétiques; and that it is far more agreeable to spend the morning hours in a pleasant walk than in anxious attention to the laying on of pigments and the dread discovery of crow's feet.

### Everybody's Opinion.

ST. MARY'S COTTAGE HOSPITAL, PLAISTOW.

"Sister Agnes" writes: I must ask you to correct a statement in your issue of The Hospital of May 12th. I have been Sister-in-Charge of this hospital since it was first started in the year 1891, and have no intention of resigning my appointment at present.

*** Our notice referred to the Matron of the Day Nursery and Hospital at Plaistow, not the Matron of the Cottage Hospital "Sister Agnes" refers to.—Ed. *T. H.*

### The Medico-Psychological Association.

COUNTY ASYLUM, MICKLEOVER.

Nursing Certificates.

At the written and oral examinations for certificates of proficiency in nursing granted by the Medico-Psychological Association of Great Britain and Ireland, held at Mickleover Asylum on May 7th and 11th, Dr. Whitcombe, medical superintendent of the Birmingham City Asylum, and ex-president of the association, being the examiner, 19 attendants, 13 men and 6 women were successful in passing the examinations, and will shortly receive certificates. Their names in alphabetical order are George Blood, George Borrill, John William Curtois, Joseph Caborn, Arthur Dean, William Davis, Frank Harrison, Charles Markham, Henry Metcalfe, John Presland, Walter Stanley, George Stenton, Walter Thorold, Sarah Bowker, Emma Merriman, Mary Ann Pearson, Mary Pidd, Julia Smith, and Charlotte Ward.

With the advance in the modern treatment of the insane and the improvement of asylums, which are now coming to be regarded more as hospitals for the cure of mental disease, and less as mere receptacles and detention houses, much more is now expected and required from attendants upon the insane than formerly.

Asylums have of late years to a certain extent taken a leaf out of the hospital book, and it is now possible for hospitals to take a leaf out of the asylum book to the extent of instituting an examination for the nursing certificate by independent examiners unconnected with the medical staff of the hospitals.

We believe that at most hospitals a certificate for three years' training in nursing is given by the medical staff, but we are not aware that there is any independent examination whatever, either written or oral, for such certificate. For example, we believe that this remark applies to our noble local institution, the Derby Royal Infirmary, the new buildings of which are shortly to be opened. It would be a fitting inauguration of such opening ceremony in July if the Derby Royal Infirmary managers could announce the institution of an independent examination, in addition to that of the medical staff, for the nursing certificate. This suggestion might be considered by the infirmary managers in connection with any arrangements they may consider necessary for increasing the visiting medical staff by the appointment of one or two assistant-physicians and assistant-surgeons, and any reorganisation of the hospital.

### Minor Appointments.

Calverley Infectious Diseases Hospital.—Mrs. T. Cooke has been appointed Matron of the above institution. She previously held the post of Matron to the Pocklington Union.

Ceylon Association for Nurses.—Nurse Rosa Shankland has been admitted as nurse to this association. She was trained at the Crumpsall Infirmary, Manchester, and subsequently undertook private nursing for the Trained Nurses' Institution at Leeds.

Nurse R. Cash has been appointed Sister at the Stockport Infirmary. She received her training at Halifax Infirmary, and has since worked at Stratford and at Burton-on-Trent. Nurse Cash has excellent testimonials.

### For Reading to the Sick.

"RESTFULNESS."

**Motto.**

Still in thy right hand carry gentle peace,
To silence envious tongues. —*Shakespear.*

**Verses.**

I do not ask, O Lord, that Thou should'st shed
Full radiance here;
Give but a ray of peace that I may tread
Without a fear!
Joy is like restless day! but peace divine
Like quiet night.
Lead me, O Lord—till perfect day shall shine
Through peace to light! —*A. Proctor.*

How strange that all
The terrors, pains, and early miseries,
Regrets, vexations, lassitudes interfused
Within my mind—should e'er have borne a part
(And that a needful part) in making up
The calm existence which is mine—when I am worthy of myself! —*Wordsworth.*

**Reading.**

No man can be happy who is not at rest in his mind. Unhappiness and unrest of mind have been the cause of most of the crimes and sins of this mortal life, and of many of its sorrows too. And it cannot be denied that there is much in this world to make one restless and disturbed. Some try to run away from it but cannot.

They find the truth of the old saying, that "They who cross the sea change the climate but not the mind." Christ Jesus came into the world to make men happy and to give them peace of mind. Hence He said, "Come unto Me all ye that labour and are heavy laden, and I will give you rest." If we want to be happy we must learn to find "rest in the Lord" and patiently wait His will. . . . This feeling of rest in the Lord is not sloth. And it is quite different from a mere easy-going temperament. St. Paul tells us to labour, to bestir ourselves, to take trouble, in order to enter into that rest. . . . It is a rest which enables us to work, not apart from God, but in harmony with God. . . . It is a rest which springs from dependence upon God, and that dependence springs from faith, and that faith springs from a knowledge of God, as revealed in Holy Scripture. God teaches us that lesson through much severe discipline; teaches us till we learn it; teaches us by showing us how our best efforts fail if they are not according to His will. The experience of learning that lesson is often long and bitter, but we cannot doubt that the result is full happiness. We are very slow to see what really is in the Bible. If we were not so slow we should see that it is the whole teaching of God's Word how to find rest. Observe he illustrations used, even in the Old Testament, to describe the life of those children of God who are willing to accept their Father's guidance. They are said to be safe under the shadow of the Rock, while the storms rage without, hidden in God's own tabernacle from tho strife of tongues. . . Think what it is to be kept safely in God's own Tabernacle, hidden privily by His own presence from the provoking of all men. If we turn to the New Testament it is full of the same lesson. It supplies the key to those apparently strange words, "Take no thought for the morrow." How marvellous is our Lord's teaching in these days of worry and fuss! How full of compassion He was to the weary, struggling crowds! How He would have felt for some of us who have long habits of evil to overcome! How he would have felt for those whose minds are filled with despair! Let us stand near Him on the mountain side and listen to Him as He cries, "Behold the fowls of the air, for they sow not, neither do they reap, nor gather into barns, yet your Heavenly Father feedeth them. Are ye not much better than they?" Take no anxious thought then, do not fret yourself. "Seek first the Kingdom of God and His righteousness," and all will be well. "Casting all your care upon Him, for He careth for you."—*Bishop of St. Andrew's "Communion of Saints."*

### The Book World for Women and Nurses.

[We invite Correspondence, Criticism, Enquiries, and Notes on Books likely to interest Women and Nurses. Address, Editor, The Hospital (Nurses' Book World), 428, Strand, W.C.]

Heavens ! A Bohemian novel. By Alois Vojtech Smilovsky. Translated by Professor Mourek and Jane Mourek. (Bliss, Sands, and Foster, London. 1894.)

It is usually a thankless task to review a translated novel; so much is necessarily spoilt by confusion of tongues and loss of style. "Heavens !" however, is a happy exception. The fact alone of its being Bohemian lends an interest to the story, for Bohemia, by language and geographical position, is so shut away from the rest of Europe that we welcome a novel from thence with at least curiosity, and the tale of "Heavens " is told in so charmingly simple and fresh a manner that curiosity must quickly give place to interest and pleasure. The hero is a Bohemian priest, who earns the name of "Heavens !" by constant repetition of that exclamation. He is absolutely unworldly and unselfish—unhesitatingly sacrificing reputation and chances of preferment by taking Jenny's child under the shelter of his roof. Jenny, the heroine, for whom the sacrifice is made, is hardly worthy of it in the eyes of the reader. She is a heartless, self-seeking young woman, who panders to the whims of a powerful baroness while trying to secure the weak-minded son for a husband. She sacrifices all to obtain her ambition, and when she fails calls upon Heavens ! her old friend, to protect her child. Her character is admirably drawn, and the whole story is set pleasantly in pictures of Bohemian life. The description of the tyrannical baroness reigning on her estate like a despotic queen reads, to our English ears, like a tale from the middle ages. We are left in no doubt as to the little moral of the story. It ends with virtue triumphant—a comfortable living for Heavens ! and the paralysis of the proud baroness; while Jenny, having been sufficiently punished for her fault, turns her back on the baron, whose tardy offer of marriage is refused by her in the spirit of "He that will not while he may," and marries a respectable merchant.

The Shen's Pigtail; and other Cues of Anglo-China Life. Pseudonym Library. (Fisher Unwin, 1894.)

There is a great freshness and novelty in these little stories. They are neither on hackneyed subjects, nor told in a hacknied manner. Anglo-China is brought home to us in a forcible manner, and the inner workings of its every-day life revealed in a singularly concise way. The social existence of its people seems to be here made clearer to us than it has been hitherto, either through the vehicle of travels or histories. It is the Chinaman as he is that we become acquainted with in this small volume of tales. The first, "The Shen's Pigtail," describes a so-called "shooting trip" in the large and elegantly-fitted houseboat of a certain Mr. Yang, who himself accompanies the party. Not to shoot, though, for the indulgence of this practice, it will be understood, as indeed of any other barbarian practice, was quite out of the question for himself. But the host would sometimes accompany his guests in their search for sport, now and again, be it admitted, indulging in a chance shot when no native was about. After a day spent in this fashion it is that the floating shooting party on these Chinese waters awaken to find themselves bound hand and foot by pirates and the boat robbed of all its goods—in especial of its guns—the why and the wherefore of the latter piracy only being explained at the conclusion of the story. The explanation only adds a dramatic finish to a very readable little narrative. The other stories are all equally pleasing to peruse, "A Little Chinese Party" being perhaps the most typical among the number of Anglo-China life as portrayed by its present biographer; but "Office Men" gives us a curious insight into the "promotion," so called, of Chinese officialism, what leads to it, and how it is held.

The Upper Berth. By F. Marion Crawford. (The Autonym Library.)

This volume is the first of a series of booklets which Mr. Fisher Unwin intends to run side by side with the already well-known and popular Pseudonym Library. The latter series are in yellow covers, the Autonyms are bound in Indian red, so that at a glance we are able to decide whether we will venture on the unknown, or choose the writing of an old acquaintance. Mr. Fisher Unwin was fortunate in starting off his new venture with Mr. Marion Crawford as captain. It may act as a pleasant surprise to some readers to find that the author of "The Upper Berth" has quite quitted the usual path, and has given the world a thrilling ghost story; a gruesome, dank tragedy of the sea, in which the horrors are graphically described. Too artistic to cheer his readers up at the expense of dramatic effect, no explanation clears the mystery away to which Mr. Crawford has introduced his readers, and so the tale might be well kept to inspire a seasonable creepiness round the Christmas fire. The "Waters of Paradise," which occupies the second half of the first volume, is also a tale full of mystery, but of a more phantastic order, and as the story ends happily, it forms a fortunate contrast to the gloom of "The Upper Berth."

### MAGAZINES.

The Nineteenth Century for May contains, among others, two articles which are of especial moment to the medical world, one of which has already been referred to in our pages. Mr. Gladstone contributes five specimens of Horace's Love Odes. "Simon Ryan the Peterite" is concluded by Dr. Jessop. "Aspects of Tennyson" are continued. In this instance it is as a humourist that Mr. Traill treats the poet's works. In doing so he recalls to our memories Fitzgerald's comment on the Poems of 1842. "Alfred," said Fitzgerald, "whatever he may think, cannot trifle. His smile is rather a grim one," and it is this very criticism which Tennyson's present biographer takes upon himself the task to disprove. For not to see the poet's humour is not to understand aright his poetry. Mr. Traill's article on this aspect of the Laureate is full of thoughtful reflection, and equals, if it does not exceed, its predecessors on the same heading.

### Books Received.

Willing and Co.
"Willing's British and Irish Press Guide," 1894.

George Allen.
"On the Care of the Dying." By Oswald Browne, M.A.

McCorquodale and Co.
"Metropolitan Asylums Board Reports for 1893."

Hirschfeld Bros.
"Domestic Hygiene." By Thomas Dutton, M.D.

Verlag von Veit and Company, Leipzig.
"Zeit Schrift für Hygiene und Infectionskrankheiten." Dr. R. Koch and Dr. C. Flügge.

Christian Evidence Society.
"The Physician's Testimony for Christ." Sir Andrew Clark, Bart., with preface by Sir Dyce Duckworth.

J. and A. Churchill.
"Uric Acid as a Factor in the Causation of Disease." By Alexander Haig, M.A., M.D.Oxon.

Swan Sonnenschein and Co.
"Disease and Race." By Fadroo.

John Bale and Sons.

"On Seborrhœa." By Joseph Frank Payne, M.D.

**Periodicals and Pamphlets.**—The Review of the Churches, Forward, London, Science Progress, Cornhill, English Illustrated Magazine, Leisure Hour, Sunday at Home, Boy's Own Paper, Girl's Own Paper, Friendly Greetings, Cottager and Artisan, Light in the Home, Child's Companion, Our Little Dots, Journal of Universal Information, The Therapist, The Popular Medical Monthly, The Planter, The Rural World, Vegetarian Reich Medicinal Anzeiger, The Sun, Charity Organisation Review, The Strand Magazine, The Picture Magazine, Medical Magazine, Monthly Register, Humanitarian, Nature versus the Devil, Homœopathic Review, Leeds Hospital Magazine, The Zenana, Amateur Gardening, Rural World, Edinburgh Missionary Society's Quarterly Paper, Medicinischer Anzeiger, Guy's Hospital Gazette, Wright's Prescription Book.

## Figures and Tables

**Figure f1:**